# A Comprehensive Review on Polyphenols of White Wine: Impact on Wine Quality and Potential Health Benefits

**DOI:** 10.3390/molecules29215074

**Published:** 2024-10-26

**Authors:** Ina Ćorković, Anita Pichler, Josip Šimunović, Mirela Kopjar

**Affiliations:** 1 Faculty of Food Technology Osijek, Josip Juraj Strossmayer University of Osijek, F. Kuhača 18, 31000 Osijek, Croatia; ina.corkovic@ptfos.hr (I.Ć.); anita.pichler@ptfos.hr (A.P.); 2 Department of Food, Bioprocessing and Nutrition Sciences, North Carolina State University, Raleigh, NC 27695, USA; simun@ncsu.edu

**Keywords:** white wine, polyphenols, quality, health benefits, bioavailability

## Abstract

Polyphenols are associated with various beneficial health effects. These compounds are present in edible plants such as fruits and vegetables, and the human body absorbs them through the consumption of foods and beverages. Wine is recognized as a rich source of these valuable compounds, and it has been well established that polyphenols present in red wine possess numerous biologically active functions related to health promotion. Therefore, most scientific research has been focused on red wine polyphenols, whereas white wine polyphenols have been neglected. This review presents the summarized information about the most abundant polyphenols in white wines, their concentration, their impact on wine quality and their potential health effects, such as neuroprotective and cardioprotective activities, antioxidant potential, antimicrobial activity and their positive effects on lipids. These findings are an effort to help compensate for the relative lack of relevant data in the scientific literature regarding white wine polyphenols.

## 1. Introduction

Wine is one of the oldest and most broadly distributed beverages throughout the world. Its chemical composition consists of alcohols, acids, sugars, proteins, minerals, polyphenols and volatile compounds. Wine polyphenols are well known for their properties related to health improvement. Additionally, the presence of different groups of polyphenols affects the wine quality; thus, through manipulation of their profile, producers can obtain a beverage with the desired characteristics [1]. It has been well established that the consumption of food polyphenols has a protective effect against cardiovascular diseases such as atherosclerosis, coronary heart disease and others. Polyphenols act as free-radical scavengers and, in this way, prevent the oxidation of low-density lipoprotein attributed to the development of atherosclerosis [2,3,4,5,6]. In a previously published study, it was reported that the consumption of wine could have health-promoting effects if it is consumed in moderation. These positive effects of bioactive components present in wine are strongly influenced by their concentration and bioavailability [1]. The health benefits of white wines are mainly attributed to the presence of polyphenols, components which have been studied in detail. However, there is still no consensus in the scientific community over which components contribute more to the overall health impact [7,8].

The health-promoting properties of wine were originally proven in the 1980s by French epidemiologists who came up with the term ‘’French paradox’’. By comparing the mortality rate from cholesterol levels and cardiovascular diseases in particular populations of the world, the positive correlation with wine consumption was observed in all the cities that participated in the investigation, except for Toulouse (France), which presented the same levels of cholesterol as Glasgow (Scotland) with lower mortality rates caused by cardiovascular diseases. This divergence between high levels of cholesterol and lower death rates was explained by the consumption of the Mediterranean diet, which is a way of eating that emphasizes fruits, vegetables, olive oil and wine. The main conclusion of this study was that moderate wine consumption ameliorated the negative outcomes for the cardiovascular system even with a diet rich in fat. It was also concluded that longer life expectancy is associated with those who consumed wine in moderation compared to those who consumed none at all or consumed it excessively [1,9,10]. There are several previously published studies dealing with the relationship between red wines and decreased oxidation of low-density lipoprotein cholesterol [11,12,13]. White wines are the ones with lower concentrations of total polyphenols, and research regarding their impact on human health has been less common. Most recently, scientific studies on the production of white wines have been directed towards the utilization of prolonged maceration in order to obtain white wines with higher contents of polyphenols, i.e., similar to those of red wines. Published studies show that white wines produced by the application of the maceration step were rich in polyphenols [2,14,15]. However, prolonged maceration can affect the sensory characteristics of white wine. An increase in polyphenol concentration causes an astringent and bitter taste, which is usually undesirable in white wines [14,16]. Additionally, prolonged maceration can affect the flavor profile of white wines due to the formation of volatile compounds [15,17]. Prolonged maceration affects not only the flavor of white wine but also color since usually these wines are characterized by orange/amber color [14].

There are different analytical methods for the determination of polyphenols in white wines, including reaction with Folin–Ciocalteu reagents and other UV-Vis spectrophotometry methods, gas chromatography-mass spectrometry analysis, liquid chromatography, high-performance liquid chromatography, capillary electrophoresis and ultra-performance liquid chromatography [18,19,20]. Additionally, various electrochemical methods such as cyclic, differential pulse and linear sweep voltammetry and spectroscopic methods such as Infrared (Fourier transform infrared spectroscopy and near-infrared spectroscopy), Fluorescence and Raman spectroscopy have been applied [20,21]. All of the above-mentioned methods are efficient, non-destructive and cost-effective. The only potential obstacle regarding these methods is their implementation in industrial processes, as their adaption is necessary to achieve their application in wineries [22].

There is still a lack of evidence to fully confirm and evaluate the biological effects of polyphenols in white wine. The polyphenols profile of white wines is complex, and their biological effects depend on the concentrations of individual polyphenols as well as on their ratios. Additionally, other parameters, such as intermolecular interactions, dissociation, ionization, matrix interference, etc., need to be included in order to perceive the complete potential of white wine polyphenols. Consequently, the biological potential of the complete mixture can be additive, synergistic, or antagonistic [23,24,25]. Evidence of their positive effects on human health could also increase the interest in exploring the possibility of their use as dietary supplements. Additional research is therefore needed to further expand knowledge in this area [26]. Data regarding the bioavailability of polyphenols is also very important for a more precise interpretation of the observed effects [27]. For a better understanding of this topic, the present review brings summarized information about the most abundant polyphenols in white wines but also their potential health effects such as neuroprotective and cardioprotective activities, antioxidant, antimicrobial activity and positive effects on lipids.

## 2. White Wine Polyphenols

There is a large diversity in the chemical structures of polyphenol compounds both in grapes and wines. This is a consequence of various varieties of grapes used in the production of wine, but these compounds can also be found in free and conjugated forms [28,29]. The used grape variety is not the only factor affecting the chemical diversity of polyphenols. Other highly important factors are grape maturity, environmental and agro-ecological conditions in vineyards and wine-making technology, including conditions during the fermentation and storage of wine [28,30]. Manipulations with grapes prior to their crushing, such as the addition of SO_2_ or ascorbic acid, as well as other operations like maceration, are also important factors influencing the polyphenol profile of wine. Conditions during the fermentation, selection of yeast strains for inoculation, malolactic fermentation, precipitation, and clarification also significantly contribute to the overall polyphenol profile of the end-product [28,31,32].

Commonly, the classification of polyphenols into various groups is generated based on the number of phenol rings that they contain in their structure and on the structural elements that are responsible for binding these rings to one another. The main groups are flavonoids, phenolic acids, stilbenes and lignans. The structural differences are caused by different reactions, such as hydroxylation, glycosylation, acylation and alkylation, which are responsible for the alternation of the basic molecule structure. The most prevalent group of polyphenols in nature are flavonoids, which are constructed of two aromatic rings. These rings are bound to one another by three carbon atoms that form an oxygenated heterocycle, and based on a variation of this heterocycle, flavonoids may be divided into flavonols, flavones, flavanones, flavanols, isoflavones and anthocyanins. Phenolic acids are divided into two types of derivatives: derivatives of benzoic acid and derivatives of cinnamic acid. The derivatives of hydroxycinnamic acids are more common than hydroxybenzoic acids. Structures of stilbenes are characterized by two phenyl moieties, which are connected by a two-carbon methylene bridge. On the other hand, lignans are diphenolic compounds that comprise a 2,3-dibenzylbutane structure, which is established through the dimerization of two cinnamic acid residues [4]. Further, the discussion of different polyphenols in various white wines will be provided.

### 2.1. Total Polyphenols of White Wines

Katalinić et al. [33] investigated the total polyphenols contents of Croatian white wine varieties Maraština, Pošip, Traminac and Graševina and compared them to the selected red wines. As was assumed, red wines contained higher amounts of total polyphenols, flavonoids and catechins compared to white wines. The average of total polyphenols in white wines was about 333 mg GAE/L, while the red wines contained an average of about 2674.5 mg GAE/L of total polyphenols, where GAE denotes gallic acid equivalents [33]. Higher concentrations of total polyphenols in red wines are the result of different operations that are used during the wine-making process, i.e., maceration, which is used as a pre-fermentative step [1]. However, for the concentration of non-flavonoid polyphenols, a significant difference between the white and red wines was not observed. The mean concentration of non-flavonoids in red wines was about 277.8 mg GAE/L, and for white wines, it was about 306 mg GAE/L. As was expected, for flavonoid phenolic compounds and catechins, red wines contained significantly higher amounts [33]. Similar results were obtained by Soyollkham et al. [34], who determined the total polyphenol content of white wines in the range from 117 to 328 mg TE/L, whereas for red wines, the determined range was from 564 to 1216 mg TE/L, where the determined values have been expressed as tannin equivalents. Total polyphenols ranging from 244 to 303 mg GAE/L for white wines were determined by Danilewicz [35]. The observed differences in the concentrations of total polyphenols are explained by the influence of the geographic origin, annual levels of precipitation, pedology and average annual temperatures [34]. Also, variety, ripening time, extraction techniques and wine-making technologies in general affect the concentration of total polyphenols in wine [36].

### 2.2. Phenolic Acids

Phenolic acids are divided into hydroxybenzoic and hydroxycinnamic acids and are usually present in wine in their free or conjugated forms. The most important representative of hydroxybenzoic acids is gallic acid. For hydroxycinnamic acids, *p*-coumaric, caffeic, ferulic, caftaric and para-coutaric acids are the most abundant representatives. In nature, hydroxycinnamic acids are often present as esters of quinic acid units or molecules of glucose [28]. Hydroxycinnamic acid esters have an important role in the browning of white wine, as these reactions usually start at the early stages of wine-making via enzymatic reactions. This technologically important process has been described elsewhere [37]. De Quirós et al. [38] applied liquid chromatography equipped with a fluorescence detector and ultraviolet/visible light detector as tools for the identification of different compounds in white wine samples, and concentrations of 11 different polyphenols were estimated. Caftaric acid was the predominant compound in all samples [38]. The same compound was the most abundant phenolic acid in Istrian Malvasia [39]. Caffeic and gallic acids are important hydroxycinnamic and hydroxybenzoic acids found in wine. These acids are important carriers of anticancerogenic activity and also possess significant antioxidant and anti-melanogenic properties. It was reported that gallic acid also has beneficial properties for the treatment of brain tumors as a result of suppression of cell viability, invasion, proliferation and angiogenesis in human glioma cells [40]. In addition to their health-promoting properties, phenolic acids act as the precursors of other bioactive molecules which can be used in food, cosmetics and therapeutic industries. Due to these discoveries, the interest in phenolic acids has lately been very high in research and development as well as in industrial applications [41]. Phenolic compounds of 35 selected white wines from the Moravian region of the Czech Republic were analyzed [42]. The order of groups according to their content in wine was as follows: flavanols > benzoic acid derivatives > cinnamic acid derivatives > flavonols > stilbenes. From the group of phenolic acids, the most common in the wines were benzoic acid derivatives > protocatechuic acid > syringic acid > 4-hydroxybenzoic acid > protocatechuic acid ethyl ester > ellagic acid > vanillic acid [42]. Concentrations of phenolic acids in white wine are presented in Table 1. 

### 2.3. Flavonoids

Generally, the group of flavonoids is divided into several subgroups such as flavones, flavonols, flavanones, flavononols, flavanes, flavanols, anthocyanidins and anthocyanins, chalcones and dihydrochalcones. Flavanols are present in white wine as flavan-3-ols, catechin and its enantiomer epicatechin. Catechin is found in very high concentrations in white wine and its presence is thought to give the wine a specific, bitter taste [28]. Flavan-3-ols such as catechin and epicatechin have anticarcinogenic, antimicrobial and antiviral properties and are beneficial in the protection of neurological health, against liver and heart diseases and in weight loss, slowing down the aging processes and protecting skin from ionizing radiation [40]. In white wines, the most important flavonols are quercetin, kaempferol and isorhamnetin. Also, they can exist in glycoside forms but in lower contents [28]. For flavonoid glycosides, occurrence, structure, position and number of sugar groups are determining factors for antioxidant activity, since it has been proven that aglycones have higher antioxidant activity than their related glycosides. Quercetin possesses antioxidant, anti-inflammatory and anti-proliferative properties. Also, it possesses chemo-preventive ability as it plays an important role during carcinogenesis from initiation to invasion and metastasis [40]. The flavonoid content of white wines is influenced by the grape composition, the extraction technique of grapes into grape juice, subsequent reactions that occur during the vinification, treatments of wine after the fermentation and wine aging [51]. Concentrations of flavonoids in different white wines are presented in Table 2.

### 2.4. Tannins

Tannins are polyphenols that determine the characteristic astringent properties of wines and are divided into two subgroups, the hydrolyzable and the non-hydrolyzable tannins, which are also called condensed tannins or proanthocyanidins. These polyphenols originate from grape tissue and are found in the must during crushing, maceration and fermentation. They are oligomers and polymers of flavan-3-ols. In white wines, type B proanthocyanidins (oligomers of flavan-3-ols) are present [28]. Concentrations of tannins in white wines were in the range from 10 to 50 mg/L, while in red wines, it was about 593 mg/L [53]. Tannins give wine its characteristic organoleptic properties. The addition of selected commercially available tannins caused the modification of the sensory properties of wines [54]. This is explained by the ability of tannins to bind directly to oral epithelial cells with an efficacy related to their polymerization degree [55]. Concentrations of tannins in different white wines are presented in Table 3.

### 2.5. Stilbenes

Resveratrol is the main representative of stilbenes present in wine. Higher concentrations of this compound are present in wines with longer maturation periods. Its concentration is significantly higher in red wines compared to white wines [28]. For comparison, the concentration of resveratrol in red wine was 1.90 mg/L, in rose wines, 0.41 mg/L, and in white wines, 0.13 mg/L [56]. Among 7 Serbian white wine varieties, the highest total content of resveratrol was found in Graševina wine, about 0.82 mg/L [57]. Viticultural and enological factors affect the concentration of resveratrol in wine. Crucial factors are grape variety, environment and the application of other techniques during wine-making [58]. As a result of its beneficial health effects, its identification and quantification are of great importance. Many analytical methods are reported for resveratrol determination; however, as the wine matrix is very complex, its analysis is very demanding [59]. Bertelli [60] reported a significant increase in resveratrol levels after the consumption of white wine that was probably caused by the recalled resveratrol tissue stored in the bloodstream, since it was proven that resveratrol can accumulate in the heart, liver and lungs after chronic administration. Originally, resveratrol was considered a toxic compound formed by higher plants as a result of infections or similar stresses like nutrient deprivation. Since the cardioprotective effects of wine were discovered, resveratrol has become the subject of numerous scientific research efforts, especially regarding its anticancer and antioxidant properties [61]. Concentrations of stilbenes in different white wines are presented in Table 4.

For a better understanding of the polyphenol composition in white wines, polyphenol components and their concentrations determined by other investigators are presented in Table 1, Table 2, Table 3 and Table 4. As different analytical methods are applied, each of the methods of analysis is noted.

### 2.6. Polyphenols of Macerated White Wines

The International Organization of Vine and Wine defined macerated white wines as “white wines derived from the alcoholic fermentation of a must in prolonged contact with grape pomace, including skins, pulp, seeds and eventually stems.’’ This technique, which is increasingly attracting the attention of producers and researchers, as well as consumers, allows the production of wines that combine characteristics of both red and white wines [15]. These wines are also called “orange wines” or “amber wines” [43]. As was already mentioned, red wines are richer in polyphenols than white wines, so efforts were made in order to increase the white wine’s polyphenol contents. Red wine production is characterized by skin maceration, so this step can be introduced in white wine production as a pre-fermentative operation. Through maceration, phenolic compounds are extracted from the grape skins, which increases polyphenols content. However, these compounds can also contribute to increased bitterness, astringency, and the browning of white wines. This means that the overall quality of the final product is changing [17,62,63,64,65]. In Table 1, Table 2, Table 3 and Table 4, in addition to the results of polyphenols content of white wines, results of white wines produced with the application of maceration as a pre-fermentative step are also presented. It is evident that the introduction of the maceration step caused an increase in some phenolic compounds which was also associated with the increase in antioxidant potential [16,43,44]. In the research of Jagatić Korenika et al. [16], it was determined that the concentrations of individual polyphenols in macerated wines depended not only on the polyphenol type but also on the type of wine. Gallic acid was determined in higher concentrations in macerated Pošip wine, while vanillic, caffeic and coutaric acids were determined in higher concentrations in macerated Škrlet wine. In both macerated wines, higher concentrations of caftaric, syringic and *p*-coumaric acids were observed. Additionally, a higher concentration of catechin was determined in macerated Pošip wine. For other individual polyphenols, there was no significant difference in concentrations between unmacerated and macerated wines [16]. Evaluating values of antioxidant activity of unmacerated and macerated Malvazija and Pošip wines by utilization of FRAP and ORAC methods, it was determined that macerated wines had higher antioxidant potential [44]. As in the above-mentioned study, authors also determined that the concentrations of individual polyphenols in macerated wines depended on both parameters, polyphenols and wine types. Gallic, protocatechuic, *p*-hydroxybenzoic, *trans*-caftaric and *p*-coumaric acids were determined in higher concentrations in macerated Malvazija wine. In macerated Pošip wine, higher concentrations of gallic, syringic, *trans*-caftaric, caffeic, ferulic and *p*-coumaric acids were observed. While in macerated Malvazija wine, higher concentrations of taxifolin and quercetin were observed, a reverse trend was observed in macerated Pošip wine. Concentrations of (+)-catechin, procyanidin B1, procyanidin B2 and *cis*-piceid were determined in higher concentrations in macerated Malvazija wine. In macerated Pošip wine, higher concentrations of procyanidin C1, resveratrol and trans-piceid were observed. Milat et al. [43] reported that values of the antioxidant activity of macerated Graševina wine, evaluated by FRAP, DPPH, ABTS and ORAC methods, were significantly higher than those of unmacerated. For unmacerated wine, values of antioxidant activity were 336.7 µmol TroloxE, 213.3 µmol TroloxE, 3037.5 µmol TroloxE and 4756.7 µmol TroloxE, respectively, while these values for macerated wine were 14362.6 µmol TroloxE, 12,145.0 µmol TroloxE, 16178.6 µmol TroloxE and 23398.3 µmol TroloxE, respectively. Additionally, they observed that macerated wine contained significantly higher concentrations of several phenolic acids (*trans*-caftaric acid, *cis*-coutaric acid, caffeic acid, *trans*-ferulic acid, gallic acid and vanillic acid), two flavonoids ((+)-catechin and (-)-epicatechin) and two tannins (procyanidn B2 and procyanidn B4).

## 3. Polyphenols and Quality of White Wines

Since wine is one of the most widely consumed beverages in the world, manufacturers and sellers need to ensure its quality [66]. The increasing interest of consumers in functional foods consequently resulted in the need for the development of chemical methods for assessing phenolic composition. In addition to the importance of their health-promoting properties, it is also essential to evaluate the composition of polyphenols as they contribute to the color, flavor, oxidative stability, astringency and bitterness of wines [67]. Polyphenols are also often used as markers of wine authenticity [68,69]. White grape skins contain lower anthocyanin content, and thus, white wines exhibit reduced levels of total phenolic and flavonoid compounds compared to red wines. Phenolic compounds represent the secondary metabolites found in grapes and wine. They have an important role as quality indicators in wine production. These compounds are crucial in defining the attributes of white wine.

### 3.1. Impact on the Mouthfeel

The mouthfeel of white wine is a combination of the perception of astringency, bitterness, hotness and viscosity, which are taste, chemosensory and tactile attributes [55,70]. All of these attributes are consequences of wine composition, particularly, phenolic compounds, ethanol, glycerol, pH, polysaccharides, dissolved carbon dioxide and peptides. Polyphenols have a wide effect on the mouthfeel since they contribute to all the above-mentioned attributes due to their structural diversity, which ensures their involvement in multiple sensory mechanisms of mouthfeel perception [55]. In model white wine systems where polyphenols extracted from white wines were added to those model wines at pH 3.3, a significant increase in astringency was observed. However, the same effect was not observed in model wines adjusted to pH 3.0 [71]. Generally, it was observed that higher polyphenols caused an increase in bitterness as well as in the viscosity of model white wines, but the effect depended on the chemical structure of polyphenols. Interactions between compounds are of high significance, and it was determined that the combined effect of polyphenols content and concentration of alcohol on astringency and bitterness was additive, meaning that alcohol directly had an effect on mentioned attributes in white wines [71]. A positive correlation was established between hydroxycinnamic acids and their derivates in perceived acidity. Even though these phenolic acids were found in low concentrations (below 30 mg/L) and are weakly acid (pKa around 4.4), results indicated that they contribute to the perception of acidity [72]. Perception of hotness on the palate and burning aftertaste in wines were correlated with the presence and concentration of flavanols. To the burning aftertaste, the presence and concentration of hydroxybenzoic acids, flavanonols and dihydroxyquercetin are also of significant relevance. Even though none of these compounds have been associated with an increase in perception of hotness, total wine phenolics at a level equivalent to that of hard pressing were able to increase the perceived hotness of lower alcohol (11.5% *v*/*v*) model wine [71]. In the study of Gawal et al. [72], the concentration of hydroxycinnamic acid was negatively related to hotness and burning aftertaste. Caftaric acid, one of the major hydroxycinnamic acids in white wines, was found to suppress the burn sensation, which is produced, by other white wine phenolics and also ethanol [73]. Full-bodied white wines are characterized by viscosity and oiliness. These characteristics can be correlated with the presence of syringic and gentisic acids, quercetin glucuronide and dihydroquercetin. Higher viscosity in white wines has been perceived in wines with higher total phenolics [74], while greater oiliness in wines with higher polyphenols concentrations and grape reaction products (enzymatically formed complex of caftaric acid and glutathione, which is abundant in wines produced by causing oxidation of juice) [73]. The importance of chemical structure can be seen from the fact that flavanols were positively correlated with the intensity of bitterness, hotness and burning aftertaste, while flavonols had the opposite effect. To improve the mouthfeel of white wine, manipulation with extraction can be helpful, i.e. minimizing the extraction of seeds rather than the extraction of skin, which can result in a better mouthfeel of the final product [72].

### 3.2. Impact on Flavor

Polyphenols can also affect the perception of volatile compounds in white wine by suppressing, accentuating or showing minimal effect on the perception of the volatile compounds, but they can also affect the volatile compound release from wine [75,76]. It is difficult to predict the effect of polyphenols on the expression of volatile compounds in white wines, but certain trends for some chemical groups have been observed over the years. Trained panelists evaluated the perception of four volatile compounds: 3-mercaptohexanol, 3-mercaptohexanol acetate, isobutyl methoxypyrazine, and ethyl decanoate (key flavor compounds of Sauvignon Blanc wine) in combination with caffeic acid, catechin and quercetin. It was concluded that the perception of isobutyl methoxypyrazine, 3-mercaptohexanol and ethyl decanoate was largely suppressed by the selected polyphenols, while the perception of 3-mercaptohexanol was accentuated by the addition of caffeic acid. A slight effect of accentuating the perception of mercaptohexanol acetate was achieved only by catechin [76]. The suppression of volatile compounds perceptions’ were probably due to the formation of noncovalent bonds between OH groups of polyphenols, and the volatile compound might have reduced its perception [77,78].

Wine model solutions with the addition of polyphenols (catechin, epicatechin, and a highly condensed tannin fraction extracted from wine) and volatile compounds (benzaldehyde, limonene, isoamyl acetate and ethyl hexanoate) have been formulated, and the effect of polyphenols on the volatility of volatile compounds was investigated [77]. It was estimated that the decrease in the volatility of all volatiles was correlated with the increase in applied concentrations of catechin. The fraction of condensed tannins caused a slight decrease in the release of benzaldehyde and a salting-out effect of limonene, but did not affect the two esters [77]. Terpenes are a complex group of compounds, which are mostly responsible for citric, floral, and balsamic flavors. The effect of polyphenols on the volatility of terpenes has been mostly observed in reconstituted real wines and model wine solutions. Generally, the release of the tested terpenoids decreased with the increase in tannin concentrations [75,79,80]. Esters are one of the most important families of compounds responsible for fruity notes in wines. For this group of compounds, it has been concluded that the effect of wine polyphenols depended on their hydrophobicity and polyphenol concentrations. Esters with lower hydrophobicity exhibited higher retention at lower polyphenol concentrations, while at higher concentrations, their release occurred. The release trend in esters with higher hydrophobicity decreased independently of polyphenol concentrations, which indicated that for these esters, hydrophobicity was the main driving force for their release from the wine matrix [75,79]. Results from another study showed that an increase in polyphenol concentrations in model wine solutions were associated with a significant and linear decrease in the volatility of 4-ethylphenol and 4-ethylguaiacol [81]. Additionally, throughout the sensory testing, it was observed that the unpleasant and characteristic “phenolic” taint, which is the consequence of the presence of 4-ethylphenols [82], was significantly higher in the samples with lower polyphenol contents. These results emphasized the significant masking effect of polyphenols on the perception of the Brett character. Understanding interactions between polyphenols and volatile compounds and the consequences of those interactions can assist winemakers in managing polyphenol levels to optimize and achieve the desirable flavor profile of wines.

### 3.3. Impact on Color

One of the major problems affecting the sensory properties and overall quality of wine is its browning. As a result of browning and oxidation reactions during processing, aging and storage, a decrease in the brightness of wines and an increase in color intensity occur [83]. In addition to its effect on sensory properties, the oxidation of polyphenols could cause changes in the redox equilibrium, and the formation of oxidized polyphenols can significantly change the antioxidant properties of wine after browning [84]. In white wines, compounds responsible for the browning are mainly hydroxycinnamic acids, such as caffeic and caftaric acids and flavanols such as catechin and epicatechin [83]. Salacha et al. [83] studied correlations between the antioxidant properties, concentration of polyphenols and browning of white wines. Results of the study showed that browning rates were positively correlated with the concentration of flavanols as well as the concentration of total polyphenols. Although white wine represents a rich source of polyphenolic compounds, one of the most significant problems is wine preservation. The antioxidant activity depends on concentration of polyphenols, which have been affected by the addition of preservatives and sulfur dioxide is the most widely used preservative in enology. It prevents oxidation and browning and also changes antioxidant potential. The antioxidant activity of white wines, as determined by the ABTS method during storage declined on average by 19% during storage. The most abundant compounds were chlorogenic acid, caffeic acid and epigallocatechin [85]. Storage temperatures under 20 °C, early SO_2_ addition and longer maceration times produce wines with the highest concentration of anthocyanins and color retention. However, the concentration of anthocyanins decreased after a few days of maceration, while the concentration of colorless polyphenols increased. To produce clear, bright wines it is necessary to use fining agents. Clarification causes the reduction in phenolic compounds involved in undesirable oxidation reactions, which also contribute to wine astringency. Fining is used to minimize the browning reactions of white wines during vinification and storage by lowering the concentration of polyphenols. Also, fining improves the organoleptic characteristics of wine. Oxidation reactions also depend on the types of used closures. Wines with screwcaps exhibited a lower decline of SO_2_ content in comparison with cork closures, which caused lower browning as a result of decreased browning reactions. In wine aging, oxygen plays a predominant role. Degradation and oxidation rates were highest in wines stored in polyethylene terephthalate bottles (due to the high oxygen permeation rate of this material), both with and without an oxygen scavenger. The addition of an oxygen scavenger caused a decrease in oxidation reactions as a result of a higher antioxidant potential. The decrease in concentration of monomeric and the increase in polymeric compound contents is suppressed in wines treated with 200 mg/L SO_2_. This treatment prevents the pathways that involve the formation of carbo-cations at the C4 position of proanthocyanidins [85].

## 4. Potential Health Benefits of White Wine Polyphenols

### 4.1. Antioxidant Potential

Oxidation processes are responsible for the initial development of cancer and cardiovascular diseases. Reactive oxygen species are formed during normal metabolism and can potentially damage proteins, lipids and DNA [86]. The human defensive system against these negative effects includes different enzymes and proteins. The intake of antioxidant substances is beneficial against reactive oxygen species [86]. Due to the importance of this property for the human body, various analytical methods for the determination of the antioxidant activity of wines have been developed. Regrettably, different methodological approaches and experimental conditions can also be associated with inaccuracies when comparing results. However, well-established methods for the determination of antioxidant activity do exist and are based on the oxidation of 1,1-diphenyl-2-picrylhydrazyl radical, 2,2′-azinobis-(3-ethylbensothiazoline)-6-sulfonic acid radical, human low-density lipoprotein and N,N-Dimethyl-p-phenylene diamine. These methods describe the scavenging capacity against a given radical and the protection from oxidation [86]. Danilewicz [35] compared FRAP, DPPH and Folin–Ciocalteu assays for the evaluation of polyphenols in white wines and examined which assay indicates their concentration the most precisely. In the DPPH assay, basic and acidic solvent impurities affect results, and thus, this assay is less robust, while the FRAP assay is more robust and evaluates the concentrations of polyphenols susceptible to oxidation more specifically. Folin–Ciocalteu assay is less selective, depending strongly on the concentration of the most oxidizable polyphenols. Polyphenols that are the most reactive with oxygen are those with pyrogallol and catechol units [35]. The antioxidant activity of wines obtained by FRAP assay was studied by Katalinić et al. [33]. The antioxidant activity of wine does not depend on only one component, as it is a complex mixture of various polyphenols. Thus, it is crucial to identify which group of polyphenols affected the antioxidant activity of wine the most. Comparing the FRAP value of pure (+)-catechin versus the calculated average values for white wines with equimolar concentrations of catechins (1000 µmol/L of wine), it was observed that white wine contained polyphenols with higher reducing ability than (+)-catechin. The authors concluded that for white wines, reducing ability was related to catechins [33]. Red wine contains a higher concentration of total polyphenols; thus, it shows higher antioxidant capacity than white wine [87]. However, important conclusions have been reached by Tekos et al. [88]. In their study, extracts obtained from two white and two red wines were analyzed for their ability to catch synthetic (DPPH^•^ and ABTS^+•^), but also endogenous (^•^OH, ^•^O2^−^) free radicals, their reducing power by determination of the ability to reduce Fe^3+^ to Fe^2+^, mutagenic and antigenotoxic properties. In all applied assays for the determination of antioxidant activity, red wine extracts showed lower IC_50_ values, except for the hydroxyl radical assays, for which one of the white wine varieties showed the highest antioxidant activity. These results support the importance of heterogeneity, which strongly affects the antioxidant properties, meaning that antioxidant activity is a result of the concentration of individual polyphenols and their intermediate interactions and chemical structures. Both white wine extracts showed positive antimutagenic and antigenotoxic properties [88].

The correlation between the concentrations of individual polyphenols and the antioxidant activity of white wines was tested in the study by Alonso et al. [89], and the results showed a high correlation between the concentration of resveratrol and the antioxidant activity. Caftaric acid and coutaric acid also showed a strong correlation, while the correlation for caffeic acid was notable [89]. In the study by De Quirós et al. [38], a good correlation between the presence of different flavanols and antioxidant activity was observed. Each polyphenol present in wine contributed to the antioxidant activity differently and it was observed that antioxidant activity was related to the chemical structure of the polyphenols present [38]. The correlation between the total polyphenols content and the antioxidant activity determined by DPPH, FRAP and CUPRAC assays in white wines from 3 varieties was also established. Thus, it was concluded that polyphenols are the main antioxidant components of white wines [90]. Paixão et al. [91] tested white wine from Portugal for antioxidant capacity by applying DPPH, ABTS and FRAP assays in order to obtain a correlation with the wine’s total polyphenols content. It has been found that the reducing power of white wines was strongly correlated with the total polyphenols content. Other authors obtained similar conclusions; there was a significant and high correlation between the antioxidant capacity and the concentration of total polyphenols for 10 tested Serbian white wines [47]. Psarra et al. [92] investigated the polyphenolic constitution of 26 different Greek white wines and studied the relation between two main categories of polyphenols, hydroxycinnamates and flavanols, with antiradical and reduction properties. It was concluded that the concentration of both groups of polyphenols exhibits broad variations. These variations could be attributed to genetic factors and the stage of grape maturation, as well as the vinification techniques. Their results showed that the contents of hydroxycinnamates and flavanols influence the reducing effects greatly. Antiradical efficiency is shown to be derived from both total hydroxycinnamate and total flavanol contents, proving that synergism is involved in those mechanisms. Both groups of mentioned polyphenols define the antioxidant properties of white wines, even though the antiradical capacity is mostly governed by the total hydroxycinnamates, most likely because of their higher content. Similar results were obtained in another study [93], where 26 commercially available white wines were analyzed for total hydroxycinnamates and non-hydroxycinnamates. It was concluded that it is important to determine the concentrations of individual polyphenols to obtain their correlation with antioxidant properties. However, antiradical activity is not only affected by individual compounds but also by the synergistic effects that occur among individual polyphenols [93]. The study by Pignatelli et al. [94] focused on the inhibition of oxidative stress by wine polyphenols. Twenty healthy subjects were given 300 mL of red or white wine per day for 15 days, and after that, urinary markers of oxidative stress (PGF-2α-III) and individual polyphenols in plasma were determined. Results showed that the level of PGF-2α-III in urine significantly decreased in subjects taking both wines. Higher plasma polyphenols were detected in plasma from red wine subjects compared to the subjects consuming white wine, although concentrations of resveratrol, caffeic acid and catechin increased after intake of both white and red wine. These support the theory that antioxidant activity depends on the synergism among polyphenols.

Rats with streptozotocin-induced diabetes were studied by Landrault et al. [95] to determine the impact of polyphenol-enriched white wine on plasma antioxidant capacity. For comparison, the effect of the same polyphenol-enriched Chardonnay from which ethanol had been removed was also determined. Diabetic rats had a much lower plasma antioxidant capacity than the non-diabetic group. Therapy with polyphenols-enriched white wine or ethanol-free derivative recovered the plasma antioxidant capacity of diabetic rats to the levels observed for healthy, non-diabetic animals. Thus, it can be concluded that the intake of polyphenols-enriched white wine or ethanol-free derivative successfully recovered the antioxidant capacity, which was decreased by diabetes. Polyphenols from polyphenols-enriched white wine play a role as antioxidants in vivo and depend on the presence of ethanol. However, polyphenols-enriched white wine leads to ethanol-independent in vivo impacts in insulin-deficient diabetes caused by oxidative stress [95].

### 4.2. Lipid Effects

One of the important factors for the elimination of cholesterol from the body is the presence of high-density lipoprotein cholesterol. Wine increases high-density lipoprotein and decreases low-density lipoprotein cholesterol levels. A study by Flechtner-Mors et al. [96] showed that a diet with 10% energy derived from white wine for 3 months caused a reduction in cholesterol, blood pressure, blood glucose, triglycerides, body weight in obese patients, percent body fat and waist circumference. Rajdl et al. [97] reported that consumption of 375 mL of white wine per day for 4 weeks caused an increase in high-density lipoproteins and paraoxonase 1 in the examined group that consisted of females only. Goldberg et al. [63] also observed that the same intake for the same period of time caused an increase in plasma high-density lipoprotein cholesterol. However, it also had a negative effect as total cholesterol and triglyceride levels were also higher [98].

Resveratrol acts both as a direct and indirect antioxidant. It can decrease the oxidation of low-density lipoproteins in humans, which are usually activated by metal ions or peroxynitrites. Different enzymes are involved in the oxidation of this lipoprotein, such as nicotinamide adenine dinucleotide phosphate oxidases, myeloperoxidase 15-lipoxygenase and hypoxanthine/xanthine oxidase. Resveratrol has a negative impact on those enzymes, and thus, the formation of reactive oxygen species is lowered. Resveratrol also affects the metabolism of omega-3 fatty acids. Moderate wine consumption increases marine omega-3 concentrations in plasma [60]. Cardiovascular diseases are often caused by the oxidation of low-density lipoprotein cholesterol and a high-fat diet that causes high levels of oxidative damage to plasma lipoproteins. It was proven that wine flavonoids act against the low-density lipoprotein oxidation [7]. In the study by Sharpe et al. [99], the effect of white wine on cholesterol, high-density lipoprotein levels and triglycerides was investigated. After consumption of 200 mL of white wine per day for 10 days, no effect was observed [99]. The oxidation process of cholesterol is usually activated by active oxygen or free radicals and, 7-ketocholesterol, 5,6-epoxycholesterol and 7-hydroxycholesterol are formed, which are products of cholesterol oxidation. These oxidation products damage the macrophage and endothelial cells in blood vessels and the cholesterol is deposited on the inside walls of blood vessels, which consequently leads to the development of cardiovascular diseases [99]. Tian et al. [13] discovered that white wine polyphenols have important anti-cholesterol-oxidation properties as Chenin Blanc and Sauvignon Blanc white wines showed no 7-ketocholesterol formation during 48 h of oxidation for white wines at a 1:10 ratio of wine to the cholesterol emulsion. For Sauvignon Blanc at a 1:10 ratio, the inhibition rate was still 100% even after 72 h of oxidation. In the same study, red wine showed a better capability to inhibit cholesterol oxidation. However, the importance of white wine polyphenols in the inhibition of cholesterol oxidation should not be neglected.

In the study by Milat et al. [100], the effects of white wine polyphenols on weight gain were tested on two age groups of mice for 4 weeks. Because the impact of alcohol on body weight may differ with age, two groups were studied: younger rats during their fast growth phase and animals close to their body weight maximum. Each group was finally divided into three subgroups: water-only drinking controls, standard white wine, and polyphenol-rich white wine-drinking animals. Polyphenols detected in those wines were gallic acid, (+)-catechin, (−)-epicatechin, procyanidin B1, and resveratrol. In both age groups, the wine-drinking animals consumed less food and gained, both weekly and overall, less weight. Furthermore, there was no difference detected between the effects of consumption of standard and polyphenol-rich white wine on body weight gain and food intake. That finding could support the conclusion that wine polyphenols are of secondary importance compared to the other constituents of wine, such as ethanol, when discussing weight gain and food intake influences.

### 4.3. Cardiovascular Health

One of the most widespread causes of death in humans is cardiovascular disease. The decrease in mortality associated with these diseases could be associated with moderate wine consumption, which means drinking one to two glasses a day. White wine has shown cardioprotective effects as a result of the presence of alcohol and antioxidant compounds, including caffeic acid, hydroxytyrosol and tyrosol [7]. As most of the studies of cardioprotective effects of wines have mostly been concentrated on red wine polyphenols, Boban et al. [101] determined the effects of moderate consumption of white wine and ethanol on male Sprague Dawley rats. These were divided into three different groups: water only, white wine or a 13% *v*/*v* ethanol/water solution. Myocardial infarction was induced by ligating the left anterior descending artery and the results showed that the survival rate was the highest in the white wine group, which was 72.2%, while the lowest rate was observed in the water-only group (47.8%). The group that consumed ethanol/water solution had a survival rate of 64.7%. It was concluded that moderate consumption of white wine can have a positive effect on survival after a myocardial infarction, and it is related to other white wine constituents, not only ethanol. Also, ethanol and polyphenols may have synergistic protective effects against cardiovascular diseases. Positive cardiovascular effects may be the result of the effects of other organs, such as protection against postprandial oxidative stress or prebiotic effects on favorable gut microbiota composition [101]. Cui et al. [102] investigated the protection provided by white wine from ischemia–reperfusion injury. Extracts of three different wines after the removal of ethanol were given orally to Sprague Dawley rats for three weeks. Their results showed that only one wine among the three different white wines had a cardioprotective effect, as improved post-ischemic ventricular recovery and reduced myocardial infarct size were both observed, compared to the control group that was given only water for the same period. The antioxidant activity of wines was tested by the chemiluminescence technique as well, and it was determined that white wines can scavenge both superoxide anions and hydroxyl radicals, but the one with the highest biological effect was the one with the highest capability to scavenge the mentioned radicals. Polyphenols detected by HPLC analysis were catechin, resveratrol, caftaric acid, tyrosol and quercetin. White wine, with the highest biological potential, contained a higher content of catechin, resveratrol, and caftaric acid compared to the other two wines [102].

Although there is still no scientific consensus on the effect of wine polyphenols on platelet functions, some studies indicated that wine intake was associated with the reduction in platelet function [59]. In the study by Pignatelli et al. [103], it was observed that 300 mL of white wine a day resulted in a higher antiplatelet activity than red wine with the same content of alcohol. The alcohol component of wine is associated with antiplatelet activity as a result of the anticoagulant properties of alcohol. It has the ability to limit blood clotting by decreasing the sticking of platelets. However, this study showed that wine polyphenols are a more important factor affecting the platelet function [103]. In the study of Lavy et al. [104], blood samples of 10 healthy males, who were supplemented with 400 mL per day of white or red wine for 2 weeks, were analyzed. The authors observed no significant impact of wine intake on plasma concentrations of blood cell and platelet counts, platelet aggregation, urea, creatinine, creatine kinase, amylase and bilirubin. However, white wine supplementation caused a temporal reduction in plasma glucose concentration from 10% to 7% after 1 week of wine supplementation and a change in prothrombin time and partial thromboplastin time, which are both important blood coagulation properties. In addition, the concentration of total carotenoids in plasma was elevated after intake of white wine from about 1.14 µg/mL to about 1.35 µg/mL [104].

The effect of white wine enriched with polyphenols from Chardonnay grapes and sparkling red wine from Pinot Noir and Chardonnay grapes on early atherosclerosis in hamsters was studied. Water was used as a control, while 12% ethanol was used to investigate the effect of alcohol. Results showed that the protective effects of white wine polyphenols against atherosclerosis are equipotential to those produced by sparkling red wine. Concentrations of cholesterol in the plasma of hamsters were lower in groups that consumed white and red wine. Additionally, an increase in the ratio apo A-1/apo B was observed. A lower concentration of cholesterol is beneficial against atherosclerosis, as hypercholesterolemia is the most important risk for cardiovascular diseases. Activities of superoxide dismutase and catalase were also increased by 38% and 16%, respectively, in the group that consumed white wine. The antioxidant activity of plasma was also increased. The aortic fatty steak area was reduced in the groups consuming white wine at 85%, while for the red wine-consuming group, it was 89%, and for ethanol, only 58% [105].

The presence of resveratrol has an important influence on the cardioprotective effects of white wine. Migration and proliferation of endothelial cells are the main processes in angiogenesis and may influence the development of atherosclerosis. Resveratrol inhibits angiogenesis induced by vascular endothelial growth factor, which is known as a neoangiogenesis stimulator [61]. The important factors affecting vasomotor functions are the vasoconstrictor endothelin-1 and vasodilator nitric oxide. By inhibiting the formation of those vasoconstrictors, resveratrol can decrease the risk of atherosclerosis development. It can also inhibit the formation of endothelin-1 activated by oxidative stress [61].

There are numerous studies [106,107,108,109] dealing with the prevention of cardiovascular diseases by polyphenols. The study by Manach et al. [27] reviewed the effect of polyphenols on atherosclerosis, antioxidant activity, lipemia, endothelial function, hemostasis and inflammation.

### 4.4. Neuroprotection

Polyphenols have the ability to protect cells from oxidative alteration and prevent the development of degenerative disorders that occur as a result of oxidative stress, such as Parkinson’s and Alzheimer’s disease. By increasing the depleted glutathione levels and succinate dehydrogenase activity and by reducing the concentration of malondialdehyde, nitric oxide and xanthine oxidase, resveratrol reduces oxidative stress [110,111]. Various endogenous neurotoxins are responsible for the development of Parkinson’s disease. It was reported that catechin has a protective role against brain injuries that occur as a result of the presence of neurotoxins. Catechin demonstrated the potential to inhibit inflammation processes and can also prevent the triggering of microglia and astrocytes associated with the formation of the mediators caused by the apoptotic death of neurons [112]. Flavonols showed the ability to protect neuronal cells against neurotoxicity that occurred as a result of oxidative stress. Extracellular accumulation of amyloid-β peptide caused neuronal loss and development of Alzheimer’s disease [113]. Protection against ischemic injury can be achieved by the application of quercetin. Phenolic acids also showed good neuroprotective properties. It was reported that amyloid-β peptide-activated toxicity and peroxynitrite-induced neuronal injury can be mitigated by caffeic acid, while ferulic acid can protect the primary neuronal cells against hydroxyl- and peroxyl-radical-mediated oxidative damage [114]. The beneficial effects of white wine polyphenols in the pathophysiological cascade associated with the neuropathology developed by 3xTg-AD mice were also recorded by Mendes et al. [115]. In the same study, the reduced accumulation of amyloid-β associated with a diet rich in white wine polyphenols was observed.

### 4.5. Antimicrobial Activity

Daglia et al. [116] studied the antibacterial activity of commercial white wine against *S. pyogenes*, a microbe that causes pharyngitis and streptococci that cause the formation of caries. It was concluded that succinic, malic, lactic, tartaric, citric and acetic acid have antibacterial activity, while wine polyphenols did not show this property. These findings confirm that white wine is also associated with improved oral health.

### 4.6. Anti-Inflammatory Properties

**Figure 1:**
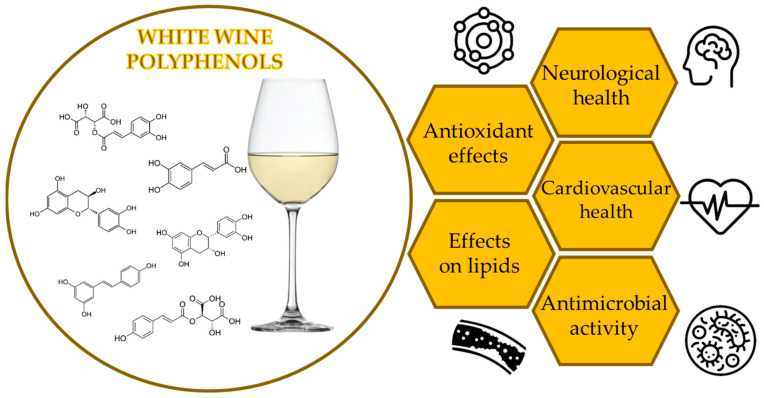
Health-promoting properties of white wine polyphenols.

Chronic inflammation is a very important factor affecting the development of many diseases, such as cardiovascular diseases, obesity, diabetes, aging, neurodegenerative diseases and the occurrence of cancers. Overall, red wine has a larger positive impact on immune functions than white wine [7]. Bertelli et al. [117] reported about the remarkable effect of white wine non-alcoholic compounds on oxidative stress. Three white wines from Italy and four white wines from Germany were tested for their content of tyrosol and caffeic acid. Results showed that pre-treatment of endothelial cells with these compounds caused a decrease in antioxidant potential in vivo. In addition to antioxidant activity, in the same study, the inflammatory response was tested as well. It was concluded that three different cytokines, interleukin-1β, interleukin-6 and tumor necrosis factor alpha, were modulated by tyrosol and caffeic acid. These cytokines are able to cause an inflammatory response and regulate the transcription of acute-phase proteins. These substances were tested combined, and a synergistic effect was noted even at lower concentrations. Migliori et al. [118] studied the effect of white wine on inflammatory markers in 10 healthy volunteers and in 10 patients with chronic kidney disease. Although white wine has lower concentrations of polyphenols than red wine, it contains simple phenols such as tyrosol and hydroxytyrosol, which are also found in extra-virgin olive oil. Results showed that plasma markers of chronic inflammation were significantly reduced in patients with chronic kidney disease during the combined consumption of white wine and olive oil. Figure 1 presents a schematic summary of potential health-promoting properties of white wine polyphenols.

### 4.7. Negative Health Effects

Wine is also an important constituent of the Mediterranean diet, which is considered beneficial for cardiovascular health. However, the risk of wine consumption is associated with its alcohol content; thus, its potential negative effects on human health, such as liver diseases, infectious diseases and non-intentional injuries, hypertension and sudden cardiac death, should also be taken into consideration [119]. Another possible negative effect of immoderate white wine intake is the occurrence of gastroesophageal reflux in healthy people. This reflux is caused by the inhibition of postprandial gastric contractions and disturbed esophageal clearance due to the increase in simultaneous failed peristalsis and contractions. To avoid this, it is important to limit the intake of white wine to 300 mL a day. All positive health effects are a consequence of moderate wine consumption, which is usually two drinks per day for men and one drink per day for women. Even the consumption of three glasses a day can cause serious negative consequences. Contraindications also appear in children, pregnant women and patients with liver disease or those who are already on pharmacological therapy [7].

## 5. Issues Regarding Bioavailability of White Wine Polyphenols

Despite numerous studies regarding polyphenols intake and lowered risk factors for the development of chronic diseases, there are distinctions in their positive effects as a result of their bioavailability [120]. To evaluate the potential biological activity of polyphenols, it is necessary to know their bioavailability. Bioavailability is defined as the fraction of an ingested nutrient or compound that reaches the systemic circulation and the target tissue where it shows its bioactivity [121]. Under the term bioavailability, two additional terms are defined. Bioaccessibility is the part of an ingested compound released from its food matrix available for absorption in the intestine, while the term bioactivity includes all the physiological effects that its consumption causes [122]. The low absorption of polyphenols and their insufficient concentrations at their cellular sites of action cause their lower in vivo bioactivity. In order to provide more precise conclusions regarding their effects on human health, there is a need to develop and implement more appropriate analytical methods to accurately measure wine polyphenols and their metabolites in plasma and urine [123].

In the study by Goldberg et al. [124], twelve healthy men were tested to compare and test the absorption of three polyphenols present in wine: resveratrol, (+)-catechin and quercetin. After consumption, the concentrations of those polyphenols were measured from the subjects’ blood. Due to the lipophilic properties and the limited solubility of tested polyphenols, their absorption was compared from three matrices in defined doses (grape juice, white wine and vegetable juice). The highest concentrations of (+)-catechin in serum were observed after intake of grape juice. Interestingly, for quercetin, its highest concentration in serum was detected after intake of wine, while for resveratrol, no significant difference was observed. Results of these experiments have shown that the absorption of polyphenols from food sources may be inefficient to the degree where, without increased amounts, effective concentrations could be systematically unachievable in humans. It was also concluded that absorption of polyphenols does not require the presence of alcohol. Water-based media and vegetable suspensions were equally efficient as wine in enabling polyphenol absorption. However, the absorption of all polyphenols is not equal, and the absorption of resveratrol is more efficient than the other two tested polyphenols. The activities of resveratrol relevant for protecting against inflammation, atherosclerosis and cancer, as shown in vitro, show a wider spectrum and seem much more potent than other polyphenols.

Nardini et al. [125] published data for the absorption of hydroxycinnamic acids and their tartaric esters from white wines in humans. Results showed that caffeic, ferulic, p-coumaric, caftaric, fertaric and coutaric acids are bioavailable to humans. They are absorbed from the gastrointestinal tract and circulate in blood, in the form of glucuronide and sulfate conjugates.

Investigation of polyphenols’ functionality in vitro showed that drinking wine before or after a meal plays an important role in bioaccessibility under the conditions of simulated gastrointestinal digestion. Drinking wine after a meal had a better bioaccessibility of polyphenols and biological activities than drinking before a meal. Interestingly, red wine has higher biological activities than white wine, but better bioaccessibility is a result of white wine consumption, especially under binge drinking [126].

**Figure 2:**
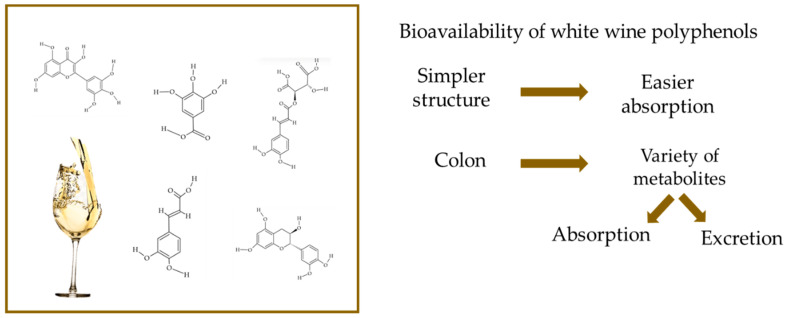
Bioavailability of white wine polyphenols.

The chemical structure of polyphenols defines the absorption of polyphenols. Studies showed that the absorption of polyphenols with plant origin occurs at about 5–10% in the small intestine. Polyphenols present in plants are mostly in the form of esters, polymers and glycosides that are not absorbed in their original structure. Prior to their absorption, these compounds need to be hydrolyzed by microbiota or enzymes. The simple structure of polyphenols causes easier absorption in the small intestine. Oligomeric and polymeric polyphenols reach the colon nearly unchanged, while monomeric and dimeric polyphenols are easily absorbed. After absorption, polyphenols are hydrolyzed and transformed into enterocytes and hepatocytes. Different water-soluble metabolites, such as methyl derivatives, glucuronides and sulfates, are formed during this process. These metabolites are distributed to tissues and in the urine, excreted in the bile in the colon and then hydrolyzed by bacterial enzymes. Polyphenols largely remain intact as they travel to the colon, where they help maintain the intestinal barrier and exhibit their beneficial properties, such as anti-inflammatory and antioxidant effects. While polyphenols are distributed throughout various tissues in the body, their majority ends up in the colon. Here, they undergo enzymatic breakdown by the gut microbiota, resulting in the production of a variety of metabolites. These metabolites can either be absorbed into the bloodstream or excreted in feces. Those absorbed are transported via the portal vein to the liver, where they may undergo further modifications like glucuronidation, methylation, or sulfation before being distributed to different tissues or eliminated through urine [127]. Stomach digestion of phenolic acids from wine was studied by Sun et al. [126]. Considering the consumed amounts, the gallic acid content increased with the higher intake during the stomach digestion step. However, the gallic acid content in the serum was only two-thirds of that found in the wines and decreased with increasing consumption. In contrast, the gallic acid content in the colon-available fraction increased with higher intake. This could be due to the total amount of gallic acid increasing while the transportation rate remained constant, leading to lower serum levels and higher colon levels. Caffeic acid, one of the main hydroxycinnamic acids in wine, showed a different trend compared to other phenolic acids. After stomach digestion, the caffeic acid content significantly increased for both red and white wines. On the other hand, chlorogenic acid showed a different trend, with its content decreasing by nearly 50% after stomach digestion. This decline might be caused by its hydrolysis into caffeic acid and quinic acid [126]. Figure 2 presents the bioavailability of white wine polyphenols.

## 6. Conclusions and Future Perspectives

There is abundant scientific evidence supporting the health benefits associated with moderate and regular wine consumption. Wines are different in their alcohol contents as well as their phytochemical contents. Although white wines contain lower concentrations of total polyphenols than red wines, their bioactivity should not be neglected. The review presents summarized information about the most abundant polyphenols in white wines, as well as their potential health effects such as antioxidant effect, cardioprotective and neuroprotective activities, and impact on wine quality. This knowledge could be useful in exploring the possibility of their use as dietary supplements. However, wine consumption should not replace the recommended pharmacotherapy or a healthy lifestyle. Future research should be focused on the bioavailability of bioactive phytochemicals of white wine, as it is the main factor limiting efficiency of their health benefits. The food and beverage industries should develop methods to enhance the bioavailability of polyphenols, possibly through food matrix modifications or encapsulation techniques, as well as better control and implementation of advanced, less damaging processing, preservation, packaging, storage and distribution technologies. Research and development of different viticultural practices, grape varieties and wine-making techniques and their effects on the polyphenols contents and their bioavailability, improvements in their positive, health-promoting bioactivities could be achieved. Additionally, potentially positive effects on human health should be further investigated by conducting clinical trials and investigations of the long-term effects of regular consumption of white wine.

## Figures and Tables

**Table 1:** Concentrations of phenolic acids and ester in different white wines.

Polyphenol	Concentration (mg/L)	Wine Type	Applied Method	Reference
Phenolic acids				
*trans*-caftaric acid	33.14 ± 0.99 5.90 ± 0.07	2 Malvazija wines from Istria, Croatia	LC-MS	[2]
	11.36 ± 0.17	Pinot Gris wine from Istria, Croatia	LC-MS	[2]
	3.42 ± 0.16	Sauvignon Blanc wine from Istria, Croatia	LC-MS	[2]
	11.54 ± 0.19 0.88 ± 0.01	2 Ribolla Gialla wines from Friuli-Venezia Giulia, Italy	LC-MS	[2]
	2.08 ± 0.03 3.49 ± 0.04	2 Tocai Friulano wines from Friuli-Venezia Giulia, Italy	LC-MS	[2]
	53.10 ± 0.80	macerated wine of bland of Malvazija, Sauvignon Blanc and Pinot Gris from Istria, Croatia	LC-MS	[2]
	33.11 ± 0.05	macerated wine of Malvazija from Istria, Croatia	LC-MS	[2]
	10.54 ± 0.05 5.71 ± 0.06	macerated 2 Ribolla Gialla wines from Friuli-Venezia Giulia, Italy	LC-MS	[2]
	4.48 ± 0.03 3.83 ± 0.04	macerated 2 Tocai Friulano wines from Friuli-Venezia Giulia, Italy	LC-MS	[2]
	25.05 ± 0.25	Graševina wine from Slavonia region, Croatia	UPLC with ESI-Q TOF	[43]
	43.60 ± 0.20	macerated Graševina wine from Slavonia region, Croatia	UPLC with ESI-Q TOF	[43]
	7.17 ± 0.20	Malvazija wine from Istria, Croatia	HPLC with DAD and FLD	[44]
	21.07 ± 0.01	Pošip wine from Dalmatia, Croatia	HPLC with DAD and FLD	[44]
	45.92 ± 0.20	macerated Malvazija wine from Istria, Croatia	HPLC with DAD and FLD	[44]
	46.70 ± 0.04	macerated Pošip wine from Dalmatia, Croatia	HPLC with DAD and FLD	[44]
	33.2–85.7	Commercial white wine from the Galicia region	LC-FID and LC-UV/Vis	[38]
	2.97 ± 0.33	Pošip from Dalmatia, Croatia	HPLC-DAD	[16]
	3.46 ± 0.26	macerated Pošip from Dalmatia, Croatia	HPLC-DAD	[16]
	8.99 ± 2.02	Škrlet from Moslavina, Croatia	HPLC-DAD	[16]
	16.96 ± 0.85	macerated Škrlet from Moslavina, Croatia	HPLC-DAD	[16]
	0–21.1	Traminer from Germany	HPLC-DAD	[45]
	0–40.2	Silvaner from Germany	HPLC-DAD	[45]
	0.5–25.9	Riesling from Germany	HPLC-DAD	[45]
	15.50–43.47	12 commercially available white wines	HPLC-DAD	[46]
	14.71 ± 1.1	Chardonnay from Serbia	HPLC-DAD	[47]
	43.53 ± 1.5	Banatski Riesling from Serbia	HPLC-DAD	[47]
	21.96 ± 1.2	Graševina from Serbia	HPLC-DAD	[47]
	7.56 ± 0.85	Traminac from Serbia	HPLC-DAD	[47]
*cis*-caftaric acid	0.71 ± 0.00	Malvazija wine from Istria, Croatia	HPLC with DAD and FLD	[44]
	0.50 ± 0.00	Pošip wine from Dalmatia, Croatia	HPLC with DAD and FLD	[44]
	0.35 ± 0.00	macerated Malvazija wine from Istria, Croatia	HPLC with DAD and FLD	[44]
	0.32 ± 0.00	macerated Pošip wine from Dalmatia, Croatia	HPLC with DAD and FLD	[44]
*trans*-coutaric acid	3.01 ± 0.04	Graševina wine from Slavonia region, Croatia	UPLC with ESI-Q TOF	[44]
	3.45 ± 0.01	macerated Graševina wine from Slavonia region, Croatia	UPLC with ESI-Q TOF	[43]
	0.20 ± 0.07	Pošip from Dalmatia, Croatia	HPLC-DAD	[16]
	0.36 ± 0.22	macerated Pošip from Dalmatia, Croatia	HPLC-DAD	[16]
	1.22 ± 0.26	Škrlet from Moslavina, Croatia	HPLC-DAD	[16]
	3.04 ± 0.76	macerated Škrlet from Moslavina, Croatia	HPLC-DAD	[16]
	0–7.5	Traminer from Germany	HPLC-DAD	[45]
	0–6.9	Silvaner from Germany	HPLC-DAD	[45]
	0.7–3.0	Riesling from Germany	HPLC-DAD	[45]
	2.66 ± 0.74	Chardonnay from Serbia	HPLC-DAD	[47]
	5.74 ± 0.76	Banatski Riesling from Serbia	HPLC-DAD	[47]
	3.71 ± 0.90	Graševina from Serbia	HPLC-DAD	[47]
	1.36 ± 0.53	Traminac from Serbia	HPLC-DAD	[47]
	1.21 ± 0.22	Semillon from Serbia	HPLC-DAD	[47]
*para*-coutaric acid	8.02 ± 0.09 5.12 ± 0.05	2 Malvazija wines from Istria, Croatia	LC-MS	[2]
	6.07 ± 0.14	Pinot Gris wine from Istria, Croatia	LC-MS	[2]
	6.43 ± 0.08	Sauvignon Blanc wine from Istria, Croatia	LC-MS	[2]
	3.36 ± 0.05 1.95 ± 0.01	2 Ribolla Gialla wines from Friuli-Venezia Giulia, Italy	LC-MS	[2]
	3.16 ± 0.03 2.58 ± 0.03	2 Tocai Friulano wines from Friuli-Venezia Giulia, Italy	LC-MS	[2]
	17.09 ± 0.02	macerated wine of bland of Malvazija, Sauvignon Blanc and Pinot Gris from Istria, Croatia	LC-MS	[2]
	4.25 ± 0.04	macerated wine of Malvazija from Istria, Croatia	LC-MS	[2]
	3.89 ± 0.06 2.65 ± 0.03	macerated 2 Ribolla Gialla wines from Friuli-Venezia Giulia, Italy	LC-MS	[2]
	2.65 ± 0.03 1.84 ± 0.03	macerated 2 Tocai Friulano wines from Friuli-Venezia Giulia, Italy	LC-MS	[2]
*cis*-coutaric acid	1.53 ± 0.01	Graševina wine from Slavonia region, Croatia	UPLC with ESI-Q TOF	[43]
	1.90 ± 0.03	macerated Graševina wine from Slavonia region, Croatia	UPLC with ESI-Q TOF	[43]
fertaric acid	1.53 ± 0.01 1.37 ± 0.02	2 Malvazija wines from Istria, Croatia	LC-MS	[2]
	1.59 ± 0.04	Pinot Gris wine from Istria, Croatia	LC-MS	[2]
	0.92 ± 0.01	Sauvignon Blanc wine from Istria, Croatia	LC-MS	[2]
	0.81 ± 0.01 0.97 ± 0.03	2 Ribolla Gialla wines from Friuli-Venezia Giulia, Italy	LC-MS	[2]
	0.92 ± 0.03 0.98 ± 0.01	2 Tocai Friulano wines from Friuli-Venezia Giulia, Italy	LC-MS	[2]
	1.77 ± 0.00	macerated wine of bland of Malvazija, Sauvignon Blanc and Pinot Gris from Istria, Croatia	LC-MS	[2]
	1.90 ± 0.05	macerated wine of Malvazija from Istria, Croatia	LC-MS	[2]
	0.93 ± 0.03 0.76 ± 0.02	macerated 2 Ribolla Gialla wines from Friuli-Venezia Giulia, Italy	LC-MS	[2]
	0.77 ± 0.01 0.79 ± 0.02	macerated 2 Tocai Friulano wines from Friuli-Venezia Giulia, Italy	LC-MS	[2]
	0.49 ± 0.07	Pošip from Dalmatia, Croatia	HPLC-DAD	[16]
	0.59 ± 0.25	macerated Pošip from Dalmatia, Croatia	HPLC-DAD	[16]
	0.36 ± 0.12	Škrlet from Moslavina, Croatia	HPLC-DAD	[16]
	0.24 ± 0.03	macerated Škrlet from Moslavina, Croatia	HPLC-DAD	[16]
2-S-glutathionylcaftaric acid	1.51 ± 0.02 1.34 ± 0.01	2 Malvazija wines from Istria, Croatia	LC-MS	[2]
	5.30 ± 0.07	Pinot Gris wine from Istria, Croatia	LC-MS	[2]
	2.30 ± 0.06	Sauvignon Blanc wine from Istria, Croatia	LC-MS	[2]
	2.96 ± 0.23 0.97 ± 0.01	2 Ribolla Gialla wines from Friuli-Venezia Giulia, Italy	LC-MS	[2]
	3.60 ± 0.02 2.46 ± 0.10	2 Tocai Friulano wines from Friuli-Venezia Giulia, Italy	LC-MS	[2]
	6.69 ± 0.07	macerated wine of bland of Malvazija, Sauvignon Blanc and Pinot Gris from Istria, Croatia	LC-MS	[2]
	1.81 ± 0.01	macerated wine of Malvazija from Istria, Croatia	LC-MS	[2]
	2.41 ± 0.04 1.55 ± 0.03	macerated 2 Ribolla Gialla wines from Friuli-Venezia Giulia, Italy	LC-MS	[2]
	1.33 ± 0.01 1.81 ± 0.01	macerated 2 Tocai Friulano wines from Friuli-Venezia Giulia, Italy	LC-MS	[2]
	1.13 ± 0.15	Chardonnay from Serbia	HPLC-DAD	[47]
	2.39 ± 0.35	Banatski Riesling from Serbia	HPLC-DAD	[47]
	1.91 ± 0.48	Traminac from Serbia	HPLC-DAD	[47]
	4.14 ± 0.52	Semillon from Serbia	HPLC-DAD	[47]
caffeic acid	3.14 ± 0.05 2.81 ± 0.03	2 Malvazija wines from Istria, Croatia	LC-MS	[2]
	1.69 ± 0.02	Pinot Gris wine from Istria, Croatia	LC-MS	[2]
	1.72 ± 0.03	Sauvignon Blanc wine from Istria, Croatia	LC-MS	[2]
	1.42 ± 0.29 -	2 Ribolla Gialla wines from Friuli-Venezia Giulia, Italy	LC-MS	[2]
	1.06 ± 0.03 2.36 ± 0.02	2 Tocai Friulano wines from Friuli-Venezia Giulia, Italy	LC-MS	[2]
	5.45 ± 0.05	macerated wine of bland of Malvazija, Sauvignon Blanc and Pinot Gris from Istria, Croatia	LC-MS	[2]
	3.20 ± 0.03	macerated wine of Malvazija from Istria, Croatia	LC-MS	[2]
	1.24 ± 0.06 0.78 ± 0.01	macerated 2 Ribolla Gialla wines from Friuli-Venezia Giulia, Italy	LC-MS	[2]
	0.68 ± 0.01 -	macerated 2 Tocai Friulano wines from Friuli-Venezia Giulia, Italy	LC-MS	[2]
	5.3	Malvazija wine from Istria, Croatia	HPLC-DAD	[48]
	3.39 ± 0.08	Graševina wine from Slavonia region, Croatia	UPLC with ESI-Q TOF	[43]
	7.34 ± 0.01	macerated Graševina wine from Slavonia region, Croatia	UPLC with ESI-Q TOF	[43]
	11.71 ± 0.00	Malvazija wine from Istria, Croatia	HPLC with DAD and FLD	[44]
	1.22 ± 0.01	Pošip wine from Dalmatia, Croatia	HPLC with DAD and FLD	[44]
	8.13 ± 0.04	macerated Malvazija wine from Istria, Croatia	HPLC with DAD and FLD	[44]
	10.61 ± 0.011	macerated Pošip wine from Dalmatia, Croatia	HPLC with DAD and FLD	[44]
	2.04 ± 0.88	Pošip from Dalmatia, Croatia	HPLC-DAD	[16]
	1.10 ± 0.09	macerated Pošip from Dalmatia, Croatia	HPLC-DAD	[16]
	0.35 ± 0.02	Škrlet from Moslavina, Croatia	HPLC-DAD	[16]
	0.78 ± 0.13	macerated Škrlet from Moslavina, Croatia	HPLC-DAD	[16]
	2.1–25.9	Traminer from Germany	HPLC-DAD	[45]
	0–13.5	Silvaner from Germany	HPLC-DAD	[45]
	0–3.9	Riesling from Germany	HPLC-DAD	[45]
	0.48–1.51	12 commercially available white wines	HPLC-DAD	[46]
	17.76–114.99	6 white wines from the Canary Islands	RP HPLC-DAD	[49]
	7.65–16.16	6 white wines from the Canary Islands	RP HPLC-DAD	[49]
	1.57 ± 0.18	Chardonnay from Serbia	HPLC-DAD	[47]
	3.39 ± 0.65	Banatski Riesling from Serbia	HPLC-DAD	[47]
	2.27 ± 0.82	Graševina from Serbia	HPLC-DAD	[47]
	7.70 ± 0.98	Traminac from Serbia	HPLC-DAD	[47]
	3.88 ± 0.80	Semillon from Serbia	HPLC-DAD	[47]
	5.0 ± 0.5	Pelješac from Dalmatia, Croatia	HPLC-DAD	[50]
*para*-coumaric acid	1.22 ± 0.00 0.68 ± 0.02	2 Malvazija wines from Istria, Croatia	LC-MS	[2]
	6.07 ± 0.14	Pinot Gris wine from Istria, Croatia	LC-MS	[2]
	1.37 ± 0.03	Sauvignon Blanc wine from Istria, Croatia	LC-MS	[2]
	0.81 ± 0.01 0.97 ± 0.03	2 Ribolla Gialla wines from Friuli-Venezia Giulia, Italy	LC-MS	[2]
	0.79 ± 0.02 0.92 ± 0.03	2 Tocai Friulano wines from Friuli-Venezia Giulia, Italy	LC-MS	[2]
	1.32 ± 0.02	macerated wine of bland of Malvazija, Sauvignon Blanc and Pinot Gris from Istria, Croatia	LC-MS	[2]
	1.5	Malvazija wine from Istria, Croatia	HPLC-DAD	[48]
	1.51 ± 0.00	Malvazija wine from Istria, Croatia	HPLC with DAD and FLD	[44]
	1.79 ± 0.00	Pošip wine from Dalmatia, Croatia	HPLC with DAD and FLD	[44]
	2.79 ± 0.00	macerated Malvazija wine from Istria, Croatia	HPLC with DAD and FLD	[44]
	3.91 ± 0.00	macerated Pošip wine from Dalmatia, Croatia	HPLC with DAD and FLD	[44]
	0.30 ± 0.04	Pošip from Dalmatia, Croatia	HPLC-DAD	[16]
	1.12 ± 0.61	macerated Pošip from Dalmatia, Croatia	HPLC-DAD	[16]
	0.26 ± 0.03	Škrlet from Moslavina, Croatia	HPLC-DAD	[16]
	0.55 ± 0.09	macerated Škrlet from Moslavina, Croatia	HPLC-DAD	[16]
	8.5–47.2	Traminer from Germany	HPLC-DAD	[45]
	0.8–33.3	Silvaner from Germany	HPLC-DAD	[45]
	0–3.7	Riesling from Germany	HPLC-DAD	[45]
	1.38–4.36	12 commercially available white wines	HPLC-DAD	[46]
	3.52–33.83	6 white wines from the Canary Islands	RP HPLC-DAD	[49]
	5.19–12.40	6 white wines from the Canary Islands	RP HPLC-DAD	[49]
	0.62 ± 0.09	Banatski Riesling from Serbia	HPLC-DAD	[47]
	0.71 ± 0.08	Graševina from Serbia	HPLC-DAD	[47]
	2.02 ± 0.65	Traminac from Serbia	HPLC-DAD	[47]
	1.72 ± 0.06	Semillon from Serbia	HPLC-DAD	[47]
*trans*-ferulic acid	0.78 ± 0.02 0.49 ± 0.02	2 Malvazija wines from Istria, Croatia	LC-MS	[2]
	1.75 ± 0.08	Pinot Gris wine from Istria, Croatia	LC-MS	[2]
	- 0.49 ± 0.02	2 Tocai Friulano wines from Friuli-Venezia Giulia, Italy	LC-MS	[2]
	1.04 ± 0.02	macerated wine of bland of Malvazija, Sauvignon Blanc and Pinot Gris from Istria, Croatia	LC-MS	[2]
	1.0	Malvazija wine from Istria, Croatia	HPLC-DAD	[48]
	3.30 ± 0.01	Graševina wine from Slavonia region, Croatia	UPLC with ESI-Q TOF	[43]
	5.20 ± 0.02	macerated Graševina wine from Slavonia region, Croatia	UPLC with ESI-Q TOF	[43]
	0.95 ± 0.00	Malvazija wine from Istria, Croatia	HPLC with DAD and FLD	[44]
	0.54 ± 0.00	Pošip wine from Dalmatia, Croatia	HPLC with DAD and FLD	[44]
	-	macerated Malvazija wine from Istria, Croatia	HPLC with DAD and FLD	[44]
	0.79 ± 0.01	macerated Pošip wine from Dalmatia, Croatia	HPLC with DAD and FLD	[44]
	0.88 ± 0.22	Pošip from Dalmatia, Croatia	HPLC-DAD	[16]
	1.01 ± 0.46	macerated Pošip from Dalmatia, Croatia	HPLC-DAD	[16]
	0.34 ± 0.06	Škrlet from Moslavina, Croatia	HPLC-DAD	[16]
	0.34 ± 0.12	macerated Škrlet from Moslavina, Croatia	HPLC-DAD	[16]
*cis*-ferulic acid	1.93 ± 0.01	Graševina wine from Slavonia region, Croatia	UPLC with ESI-Q TOF	[43]
	2.81 ± 0.03	macerated Graševina wine from Slavonia region, Croatia	UPLC with ESI-Q TOF	[43]
gallic acid	5.65 ± 0.10 -	2 Malvazija wines from Istria, Croatia	LC-MS	[2]
	1.75 ± 0.08	Sauvignon Blanc wine from Istria, Croatia	LC-MS	[2]
	3.24 ± 0.13 0.96 ± 0.04	2 Ribolla Gialla wines from Friuli-Venezia Giulia, Italy	LC-MS	[2]
	3.66 ± 0.07 3.06 ± 0.05	2 Tocai Friulano wines from Friuli-Venezia Giulia, Italy	LC-MS	[2]
	13.47 ± 0.06	macerated wine of bland of Malvazija, Sauvignon Blanc and Pinot Gris from Istria, Croatia	LC-MS	[2]
	19.38 ± 0.12	macerated wine of Malvazija from Istria, Croatia	LC-MS	[2]
	11.57 ± 0.12 8.30 ± 0.07	macerated 2 Ribolla Gialla wines from Friuli-Venezia Giulia, Italy	LC-MS	[2]
	7.56 ± 0.10 9.77 ± 0.08	macerated 2 Tocai Friulano wines from Friuli-Venezia Giulia, Italy	LC-MS	[2]
	2.18 ± 0.01	Graševina wine from Slavonia region, Croatia	UPLC with ESI-Q TOF	[43]
	20.69 ± 0.03	macerated Graševina wine from Slavonia region, Croatia	UPLC with ESI-Q TOF	[43]
	28.53 ± 0.01	Malvazija wine from Istria, Croatia	HPLC with DAD and FLD	[44]
	3.08 ± 0.01	Pošip wine from Dalmatia, Croatia	HPLC with DAD and FLD	[44]
	4.64 ± 0.02	macerated Malvazija wine from Istria, Croatia	HPLC with DAD and FLD	[44]
	30.93 ± 0.03	macerated Pošip wine from Dalmatia, Croatia	HPLC with DAD and FLD	[44]
	0.45 ± 0.11	Pošip from Dalmatia, Croatia	HPLC-DAD	[16]
	1.26 ± 0.11	macerated Pošip from Dalmatia, Croatia	HPLC-DAD	[16]
	2.57 ± 1.20	Škrlet from Moslavina, Croatia	HPLC-DAD	[16]
	2.21 ± 0.24	macerated Škrlet from Moslavina, Croatia	HPLC-DAD	[16]
	0–2.8	Traminer from Germany	HPLC-DAD	[45]
	0–14.8	Silvaner from Germany	HPLC-DAD	[45]
	5.10–9.89	12 commercially available white wines	HPLC-DAD	[46]
	0–30.95	6 white wines from the Canary Islands	RP HPLC-DAD	[49]
	8.41–35.17	12 white wines from Madeira Island	RP HPLC-DAD	[49]
	2.4 ± 0.2	Traminac from Slavonia, Croatia	HPLC-DAD	[50]
	8.4 ± 0.3	Pelješac from Dalmatia, Croatia	HPLC-DAD	[50]
	0.7 ± 0.2	Malvazija from Istria, Croatia	HPLC-DAD	[50]
protocatechuic acid	6.4	Malvazija wine from Istria, Croatia	HPLC-DAD	[48]
	0.64 ± 0.02	Malvazija wine from Istria, Croatia	HPLC with DAD and FLD	[44]
	1.02 ± 0.17	Pošip wine from Dalmatia, Croatia	HPLC with DAD and FLD	[44]
	4.56 ± 0.05	macerated Malvazija wine from Istria, Croatia	HPLC with DAD and FLD	[44]
	1.03 ± 0.04	macerated Pošip wine from Dalmatia, Croatia	HPLC with DAD and FLD	[44]
vanillic acid	4.7	Malvazija wine from Istria, Croatia	HPLC-DAD	[48]
	0.70 ± 0.03	Pošip from Dalmatia, Croatia	HPLC-DAD	[16]
	0.50 ± 0.27	macerated Pošip from Dalmatia, Croatia	HPLC-DAD	[16]
	0.46 ± 0.19	Škrlet from Moslavina, Croatia	HPLC-DAD	[16]
	2.33 ± 0.11	macerated Škrlet from Moslavina, Croatia	HPLC-DAD	[16]
syringic acid	0.7	Malvazija wine from Istria, Croatia	HPLC-DAD	[48]
	2.60 ± 0.01	Graševina wine from Slavonia region, Croatia	UPLC with ESI-Q TOF	[43]
	20.83 ± 0.23	macerated Graševina wine from Slavonia region, Croatia	UPLC with ESI-Q TOF	[43]
	0.43 ± 0.00	Malvazija wine from Istria, Croatia	HPLC with DAD and FLD	[44]
	0.14 ± 0.01	Pošip wine from Dalmatia, Croatia	HPLC with DAD and FLD	[44]
	0.16 ± 0.00	macerated Malvazija wine from Istria, Croatia	HPLC with DAD and FLD	[44]
	0.28 ± 0.00	macerated Pošip wine from Dalmatia, Croatia	HPLC with DAD and FLD	[44]
	0.56 ± 0.09	Pošip from Dalmatia, Croatia	HPLC-DAD	[16]
	0.85 ± 0.07	macerated Pošip from Dalmatia, Croatia	HPLC-DAD	[16]
	0.09 ± 0.02	Škrlet from Moslavina, Croatia	HPLC-DAD	[16]
	0.20 ± 0.10	macerated Škrlet from Moslavina, Croatia	HPLC-DAD	[16]
*para*-hydroxybenzoic acid	0.67 ± 0.01	Malvazija wine from Istria, Croatia	HPLC with DAD and FLD	[44]
	0.29 ± 0.01	Pošip wine from Dalmatia, Croatia	HPLC with DAD and FLD	[44]
	1.48 ± 0.05	macerated Malvazija wine from Istria, Croatia	HPLC with DAD and FLD	[44]
	0.23 ± 0.01	macerated Pošip wine from Dalmatia, Croatia	HPLC with DAD and FLD	[44]
	0.87 ± 0.11	Pošip from Dalmatia, Croatia	HPLC-DAD	[16]
	0.55 ± 0.03	macerated Pošip from Dalmatia, Croatia	HPLC-DAD	[16]
	3.24 ± 0.38	Škrlet from Moslavina, Croatia	HPLC-DAD	[16]
	2.88 ± 0.57	macerated Škrlet from Moslavina, Croatia	HPLC-DAD	[16]
Ester				
caffeic acid ethyl ester	1.31 ± 0.01 1.46 ± 0.02	2 Malvazija wines from Istria, Croatia	LC-MS	[2]
	0.85 ± 0.04	Pinot Gris wine from Istria, Croatia	LC-MS	[2]
	0.96 ± 0.01	Sauvignon Blanc wine from Istria, Croatia	LC-MS	[2]
	0.71 ± 0.12 -	2 Ribolla Gialla wines from Friuli-Venezia Giulia, Italy	LC-MS	[2]
	0.62 ± 0.00 0.88 ± 0.02	2 Tocai Friulano wines from Friuli-Venezia Giulia, Italy	LC-MS	[2]
	2.09 ± 0.02	macerated wine of bland of Malvazija, Sauvignon Blanc and Pinot Gris from Istria, Croatia	LC-MS	[2]
	1.51 ± 0.01	macerated wine of Malvazija from Istria, Croatia	LC-MS	[2]
	0.66 ± 0.00 0.62 ± 0.01	macerated 2 Ribolla Gialla wines from Friuli-Venezia Giulia, Italy	LC-MS	[2]
	0.64 ± 0.01 0.61 ± 0.01	macerated 2 Tocai Friulano wines from Friuli-Venezia Giulia, Italy	LC-MS	[2]

**Table 2:** Concentrations of flavonoids in different white wines.

Polyphenol	Concentration (mg/L)	Wine Type	Applied Method	Reference
(+)-catechin	6.84 ± 0.56 -	2 Malvazija wines from Istria, Croatia	LC-MS	[2]
	7.30 ± 0.26	Pinot Gris wine from Istria, Croatia	LC-MS	[2]
	21.35 ± 0.07	macerated wine of bland of Malvazija, Sauvignon Blanc and Pinot Gris from Istria, Croatia	LC-MS	[2]
	11.46 ± 0.14	macerated wine of Malvazija from Istria, Croatia	LC-MS	[2]
	15.61 ± 0.28 9.36 ± 0.13	macerated 2 Ribolla Gialla wines from Friuli-Venezia Giulia, Italy	LC-MS	[2]
	4.06 ± 0.25 13.81 ± 0.10	macerated 2 Tocai Friulano wines from Friuli-Venezia Giulia, Italy	LC-MS	[2]
	2.9	Malvazija wine from Istria, Croatia	HPLC-DAD	[48]
	5.18 ± 0.10	Graševina wine from Slavonia region, Croatia	UPLC with ESI-Q- TOF	[43]
	64.85 ± 1.30	macerated Graševina wine from Slavonia region, Croatia	UPLC with ESI-Q- TOF	[43]
	2.96 ± 0.32	Malvazija wine from Istria, Croatia	HPLC with DAD and FLD	[44]
	8.08 ± 0.30	Pošip wine from Dalmatia, Croatia	HPLC with DAD and FLD	[44]
	5.06 ± 0.10	macerated Malvazija wine from Istria, Croatia	HPLC with DAD and FLD	[44]
	6.16 ± 0.79	macerated Pošip wine from Dalmatia, Croatia	HPLC with DAD and FLD	[44]
	7.2–15.6	8 commercial white wine from the Galicia region	LC-FID and LC-UV/Vis	[38]
	1.33 ± 0.14	Pošip from Dalmatia, Croatia	HPLC-DAD	[16]
	2.03 ± 0.10	macerated Pošip from Dalmatia, Croatia	HPLC-DAD	[16]
	1.80 ± 0.44	Škrlet from Moslavina, Croatia	HPLC-DAD	[16]
	2.36 ± 0.11	macerated Škrlet from Moslavina, Croatia	HPLC-DAD	[16]
	0–26.3	Silvaner from Germany	HPLC-DAD	[45]
	25.29–42.05	4 commercial white wines from Romania	HPLC-DAD-ESI(+)MS	[52]
	1.3 ± 0.2	Traminac from Slavonia, Croatia	HPLC-DAD	[50]
	4.1 ± 0.2	Pelješac from Dalmatia, Croatia	HPLC-DAD	[50]
(-)-epicatechin	5.16 ± 0.19 3.41 ± 0.02	2 Malvazija wines from Istria, Croatia	LC-MS	[2]
	8.09 ± 0.23	Pinot Gris wine from Istria, Croatia	LC-MS	[2]
	4.05 ± 0.22 -	2 Tocai Friulano wines from Friuli-Venezia Giulia, Italy	LC-MS	[2]
	7.9	Malvazija wine from Istria, Croatia	HPLC-DAD	[48]
	13.70 ± 0.06	macerated wine of bland of Malvazija, Sauvignon Blanc and Pinot Gris from Istria, Croatia	LC-MS	[2]
	12.01 ± 0.06	macerated wine of Malvazija from Istria, Croatia	LC-MS	[2]
	8.63 ± 0.15 3.13 ± 0.11	macerated 2 Ribolla Gialla wines from Friuli-Venezia Giulia, Italy	LC-MS	[2]
	3.85 ± 0.19 7.09 ± 0.03	macerated 2 Tocai Friulano wines from Friuli-Venezia Giulia, Italy	LC-MS	[2]
	1.16 ± 0.02	Graševina wine from Slavonia region, Croatia	UPLC with ESI-Q- TOF	[43]
	42.52 ± 0.09	macerated Graševina wine from Slavonia region, Croatia	UPLC with ESI-Q- TOF	[43]
	3.40 ± 0.26	Malvazija wine from Istria, Croatia	HPLC with DAD and FLD	[44]
	3.61 ± 0.09	Pošip wine from Dalmatia, Croatia	HPLC with DAD and FLD	[44]
	0.99 ± 0.01	macerated Malvazija wine from Istria, Croatia	HPLC with DAD and FLD	[44]
	2.64 ± 0.21	macerated Pošip wine from Dalmatia, Croatia	HPLC with DAD and FLD	[44]
	2.6–28.4	8 commercial white wine from the Galicia region	LC-FID and LC-UV/Vis	[38]
	0.30 ± 0.18	Pošip from Dalmatia, Croatia	HPLC-DAD	[16]
	0.23 ± 0.02	macerated Pošip from Dalmatia, Croatia	HPLC-DAD	[16]
	0.35 ± 0.03	Škrlet from Moslavina, Croatia	HPLC-DAD	[16]
	0.29 ± 0.05	macerated Škrlet from Moslavina, Croatia	HPLC-DAD	[16]
	0–10.3	Silvaner from Germany	HPLC-DAD	[45]
	0–6.4	Riesling from Germany	HPLC-DAD	[45]
	0–89.36	6 white wines from the Canary Island	RP HPLC-DAD	[49]
rutin	4.4–33.6	8 commercial white wine from the Galicia region	LC-FID and LC-UV/Vis	[38]
myricetin	1.6–4.5	8 commercial white wine from the Galicia region	LC-FID and LC-UV/Vis	[38]
quercetin	1.4–10.3	Commercial white wine from the Galicia region	LC-FID and LC-UV/Vis	[38]
	0–30.53	6 white wines from the Canary Islands	RP HPLC-DAD	[49]
	0–20.18	6 white wines from the Canary Islands	RP HPLC-DAD	[49]
	4.1 ± 0.4	Pelješac from Dalmatia, Croatia	HPLC-DAD	[50]
	0.17 ± 0.00	Malvazija wine from Istria, Croatia	HPLC with DAD and FLD	[44]
	2.37 ± 0.01	Pošip wine from Dalmatia, Croatia	HPLC with DAD and FLD	[44]
	0.78 ± 0.04	macerated Malvazija wine from Istria, Croatia	HPLC with DAD and FLD	[44]
	1.21 ± 0.00	macerated Pošip wine from Dalmatia, Croatia	HPLC with DAD and FLD	[44]
quercetin-3-glucoside	0.28 ± 0.03	Pošip from Dalmatia, Croatia	HPLC-DAD	[16]
	0.30 ± 0.06	macerated Pošip from Dalmatia, Croatia	HPLC-DAD	[16]
	0.25 ± 0.02	Škrlet from Moslavina, Croatia	HPLC-DAD	[16]
	0.28 ± 0.04	macerated Škrlet from Moslavina, Croatia	HPLC-DAD	[16]
	5.26–6.37	4 commercial white wines from Romania	HPLC-DAD-ESI(+)MS	[52]
taxifolin	0.38 ± 0.00	Malvazija wine from Istria, Croatia	HPLC with DAD and FLD	[44]
	0.90 ± 0.01	Pošip wine from Dalmatia, Croatia	HPLC with DAD and FLD	[44]
	0.50 ± 0.00	macerated Malvazija wine from Istria, Croatia	HPLC with DAD and FLD	[44]
	0.70 ± 0.01	macerated Pošip wine from Dalmatia, Croatia	HPLC with DAD and FLD	[44]
apigenin	0.2–0.9	8 commercial white wine from the Galicia region	LC-FID and LC-UV/Vis	[38]
kaempferol	0.6–7.4	8 commercial white wine from the Galicia region	LC-FID and LC-UV/Vis	[38]
	0–0.91	12 commercially available white wines	HPLC-DAD	[46]
	0–4.24	4 commercial white wines from Romania	HPLC-DAD-ESI(+)MS	[52]
	0.4 ± 0.2	Pelješac from Dalmatia, Croatia	HPLC-DAD	[50]

**Table 3:** Concentrations of tannins in different white wines.

Polyphenol	Concentration (mg/L)	Wine Type	Applied Method	Reference
procyanidin B1	12–25.2	8 commercial white wine from the Galicia region	LC-FID and LC-UV/Vis	[38]
	1.19 ± 0.25	Pošip from Dalmatia, Croatia	HPLC-DAD	[16]
	1.07 ± 0.03	macerated Pošip from Dalmatia, Croatia	HPLC-DAD	[16]
	0.34 ± 0.08	Škrlet from Moslavina, Croatia	HPLC-DAD	[16]
	0.50 ± 0.08	macerated Škrlet from Moslavina, Croatia	HPLC-DAD	[16]
	2.73 ± 0.13	Graševina wine from Slavonia region, Croatia	UPLC with ESI-Q TOF	[43]
	37.10 ± 0.38	macerated Graševina wine from Slavonia region, Croatia	UPLC with ESI-Q TOF	[43]
	0.56 ± 0.07	Malvazija wine from Istria, Croatia	HPLC with DAD and FLD	[44]
	1.89 ± 0.19	Pošip wine from Dalmatia, Croatia	HPLC with DAD and FLD	[44]
	0.98 ± 0.09	macerated Malvazija wine from Istria, Croatia	HPLC with DAD and FLD	[44]
	0.94 ± 0.27	macerated Pošip wine from Dalmatia, Croatia	HPLC with DAD and FLD	[44]
procyanidin B2	5.1–30.4	8 commercial white wine from the Galicia region	LC-FID and LC-UV/Vis	[38]
	0–6.7	Silvaner from Germany	HPLC-DAD	[46]
	1.17 ± 0.01	Graševina wine from Slavonia region, Croatia	UPLC with ESI-Q TOF	[43]
	9.30 ± 0.02	macerated Graševina wine from Slavonia region, Croatia	UPLC with ESI-Q TOF	[43]
	0.38 ± 0.05	Malvazija wine from Istria, Croatia	HPLC with DAD and FLD	[44]
	0.96 ± 0.08	Pošip wine from Dalmatia, Croatia	HPLC with DAD and FLD	[44]
	0.53 ± 0.06	macerated Malvazija wine from Istria, Croatia	HPLC with DAD and FLD	[44]
	0.50 ± 0.08	macerated Pošip wine from Dalmatia, Croatia	HPLC with DAD and FLD	[44]
procyanidin B3	4.48 ± 1.08	Pošip from Dalmatia, Croatia	HPLC-DAD	[16]
	5.15 ± 0.84	macerated Pošip from Dalmatia, Croatia	HPLC-DAD	[16]
	2.26 ± 0.08	Škrlet from Moslavina, Croatia	HPLC-DAD	[16]
	0.50 ± 0.08	macerated Škrlet from Moslavina, Croatia	HPLC-DAD	[16]
	2.88 ± 0.41	Graševina wine from Slavonia region, Croatia	UPLC with ESI-Q TOF	[43]
	1.14 ± 0.01	macerated Graševina wine from Slavonia region, Croatia	UPLC with ESI-Q TOF	[43]
	3.60 ± 0.01	Malvazija wine from Istria, Croatia	HPLC with DAD and FLD	[44]
	4.68 ± 0.03	Pošip wine from Dalmatia, Croatia	HPLC with DAD and FLD	[44]
	1.14 ± 0.07	macerated Malvazija wine from Istria, Croatia	HPLC with DAD and FLD	[44]
	1.58 ± 0.07	macerated Pošip wine from Dalmatia, Croatia	HPLC with DAD and FLD	[44]
procyanidin B4	2.66 ± 0.10	Graševina wine from Slavonia region, Croatia	UPLC with ESI-Q TOF	[43]
	22.93 ± 0.26	macerated Graševina wine from Slavonia region, Croatia	UPLC with ESI-Q TOF	[43]
procyanidin C1	0.36 ± 0.01	Malvazija wine from Istria, Croatia	HPLC with DAD and FLD	[44]
	0.29 ± 0.01	Pošip wine from Dalmatia, Croatia	HPLC with DAD and FLD	[44]
	0.35 ± 0.01	macerated Malvazija wine from Istria, Croatia	HPLC with DAD and FLD	[44]
	0.42 ± 0.01	macerated Pošip wine from Dalmatia, Croatia	HPLC with DAD and FLD	[44]

**Table 4:** Concentrations of stilbenes in different white wines.

Polyphenol	Concentration (mg/L)	Wine Type	Applied Method	Reference
*trans*-resveratrol	0.61 ± 0.02 0.46 ± 0.02	2 Malvazija wines from Istria, Croatia	LC-MS	[2]
	0.37 ± 0.01	Pinot Gris wine from Istria, Croatia	LC-MS	[2]
	1.95 ± 0.01	macerated wine of bland of Malvazija, Sauvignon Blanc and Pinot Gris from Istria, Croatia	LC-MS	[2]
	1.18 ± 0.02	macerated wine of Malvazija from Istria, Croatia	LC-MS	[2]
	0.79 ± 0.02 0.63 ± 0.00	macerated 2 Ribolla Gialla wines from Friuli-Venezia Giulia, Italy	LC-MS	[2]
	0.49 ± 0.01 0.63 ± 0.00	macerated 2 Tocai Friulano wines from Friuli-Venezia Giulia, Italy	LC-MS	[2]
	0.12 ± 0.00	Malvazija wine from Istria, Croatia	HPLC with DAD and FLD	[44]
	0.12 ± 0.00	Pošip wine from Dalmatia, Croatia	HPLC with DAD and FLD	[44]
	0.12 ± 0.01	macerated Malvazija wine from Istria, Croatia	HPLC with DAD and FLD	[44]
	0.17 ± 0.00	macerated Pošip wine from Dalmatia, Croatia	HPLC with DAD and FLD	[44]
	1.1–4.1	8 commercial white wine from the Galicia region	LC-FID and LC-UV/Vis	[38]
	0.19 ± 0.03	Pošip from Dalmatia, Croatia	HPLC-DAD	[16]
	0.20 ± 0.06	macerated Pošip from Dalmatia, Croatia	HPLC-DAD	[16]
	0.40 ± 0.14	Škrlet from Moslavina, Croatia	HPLC-DAD	[16]
	0.44 ± 0.04	macerated Škrlet from Moslavina, Croatia	HPLC-DAD	[16]
	0–6.29	6 white wines from the Canary Islands	RP HPLC-DAD	[49]
	0–3.91	6 white wines from the Canary Islands	RP HPLC-DAD	[49]
	0.67–1.46	4 commercial white wines from Romania	HPLC-DAD-ESI(+)MS	[49]
	0.8 ± 0.2	Pelješac from Dalmatia, Croatia	HPLC-DAD	[50]
	0.1 ± 0.0	Malvazija from Istria, Croatia	HPLC-DAD	[50]
piceatannol	0.09 ± 0.01	Malvazija wine from Istria, Croatia	HPLC with DAD and FLD	[44]
	0.10 ± 0.02	Pošip wine from Dalmatia, Croatia	HPLC with DAD and FLD	[44]
	0.08 ± 0.00	macerated Malvazija wine from Istria, Croatia	HPLC with DAD and FLD	[44]
	0.06 ± 0.00	macerated Pošip wine from Dalmatia, Croatia	HPLC with DAD and FLD	[44]
*cis*-piceid	0.18 ± 0.00	Malvazija wine from Istria, Croatia	HPLC with DAD and FLD	[44]
	0.39 ± 0.03	Pošip wine from Dalmatia, Croatia	HPLC with DAD and FLD	[44]
	0.30 ± 0.02	macerated Malvazija wine from Istria, Croatia	HPLC with DAD and FLD	[44]
	0.40 ± 0.01	macerated Pošip wine from Dalmatia, Croatia	HPLC with DAD and FLD	[44]
*trans*-piceid	0.35 ± 0.08	Pošip wine from Dalmatia, Croatia	HPLC with DAD and FLD	[44]
	0.85 ± 0.08	macerated Pošip wine from Dalmatia, Croatia	HPLC with DAD and FLD	[44]

## Data Availability

Data contained within manuscript.
